# Supplementary material for: A Comparative Study on the Adipogenic Differentiation of Mesenchymal Stem/Stromal Cells in 2D and 3D Culture
Source: Cells. 2022 Apr 13;11(8):1313. doi: 10.3390/cells11081313 (PMC9029885; doi:10.3390/cells11081313)
Supplement: Supplementary file 1 [file cells-11-01313-s001.zip › cells-1644029-supplementary.pdf]

Table S1. Quantification of a subset of adipokines in cell culture supernatants and lysates from unstimulated (US) and adipogenic (AS) stimulated 2D and 3D cultures (multiplex assay, biological replicates: $n = 4$, technical replicates were involved in the calculations, represented as median and the respective minimum (Min) and maximum (Max) value, in pg/10^4 cells, in descending order depending on the amount detected, with decimal place in the single digit range).

Analyte			Supernatant						Lysate					
			2D			3D			2D			3D		
			Median	Min	Max	Median	Min	Max	Median	Min	Max	Median	Min	Max
Adiponectin	Day 7	US	0	0	20	0	0	0	0	0	0	0	0	0
		AS	17923	12191	26804	53454	1374	96499	1306	417	2580	1880	434	7972
	Day 14	US	0	0	12	0	0	0	0	0	0	0	0	0
		AS	38056	17953	60867	150746	36321	185808	2821	323	6009	1728	1450	5609
PAI-1	Day 7	US	21983	14760	27482	3809	1.5	5819	1547	826	3071	146	66	260
		AS	22154	18121	29932	2435	2255	23690	366	201	409	107	45	1232
	Day 14	US	12219	10538	17821	3702	2733	6133	1545	1053	1939	177	77	270
		AS	20027	16821	25421	2791	2073	5410	361	171	405	98	60	266
IL-6	Day 7	US	4010	2943	5947	601	0	855	34	7.9	92	11	1.9	39
		AS	1006	688	1304	51	26	665	1.7	1.2	2.3	6.9	0.4	30
	Day 14	US	2561	2108	3551	485	186	842	289	104	515	5.3	0.8	19
		AS	423	305	612	36	25	156	27	13	57	3.6	0.3	7.5
MCP-1	Day 7	US	3565	2958	5402	522	1.4	724	16	7.1	22	5.7	1.4	16
		AS	1166	1107	1548	59	45	882	8.4	6.2	8.8	1.5	0.9	36
	Day 14	US	2988	1523	5519	305	158	539	88	19	207	2.8	0.9	4.2
		AS	598	448	793	73	47	172	14	11	25	1.0	0.6	1.8
IL-8	Day 7	US	276	153	370	81	0	155	0	0	0.1	4.1	0.5	28
		AS	86	64	127	22	12	285	0.1	0	1.0	1.9	0.3	18
	Day 14	US	418	309	498	40	19	59	1.6	1.1	9.2	1.8	0	12
		AS	71	59	83	20	12	109	3.6	2.2	4.5	1.2	0.3	7.7
HGF	Day 7	US	62	25	192	305	0	442	3.1	0	25	149	96	236

		AS	261	163	675	140	52	1544	27	7.5	41	53	18	656
	Day 14	US	181	56	349	232	57	491	18	2.3	40	154	53	262
		AS	243	137	427	49	9.1	435	21	17	37	16	8.4	92
**Leptin**	Day 7	US	2.5	0	11	0	0	0	0	0	0	0	0	0
		AS	20	12	39	0	0	0	0	0	0	0	0	0
	Day 14	US	15	4.2	29	0	0	0	0	0	0	0	0	0
		AS	13	3.4	34	0.7	0	6.3	0	0	0	0	0	0
**Resistin**	Day 7	US	0.5	0	1.8	0.7	0	1.3	1.7	0.2	12	0	0	0
		AS	2.5	0	3.7	0.6	0	4.1	0.3	0	2.4	0	0	0
	Day 14	US	0.1	0	0.8	0.3	0	1.0	0	0	0	0	0	15
		AS	1.8	0	4.3	0	0	1.8	0	0	0	0	0	0
**NGF**	Day 7	US	1.4	0.9	1.6	0.1	0	0.1	0	0	0	0	0	0
		AS	1.0	0.7	1.6	0.1	0	0.6	0	0	0	0	0	0
	Day 14	US	1.0	0.7	1.8	0.1	0	0.2	0.1	0	0.2	0	0	0
		AS	0.8	0.5	1.1	0.1	0.1	0.1	0	0	0	0	0	0.1
**IL-1β**	Day 7	US	0.1	0.1	0.3	0.1	0	0.1	0	0	0	0	0	0
		AS	0.3	0.1	0.5	0	0	0.7	0	0	0	0	0	0
	Day 14	US	0.1	0	0.1	0.1	0	0.1	0	0	0	0	0	0
		AS	0.3	0.1	0.3	0.1	0	0.1	0	0	0	0	0	0
**TNF**	Day 7	US	0	0	0.2	0	0	0	0	0	0	0	0	0
		AS	0.2	0	0.2	0	0	0.5	0	0	0	0	0	0
	Day 14	US	0	0	0.1	0	0	0.1	0	0	0	0	0	0
		AS	0.1	0	0.2	0	0	0	0	0	0	0	0	0

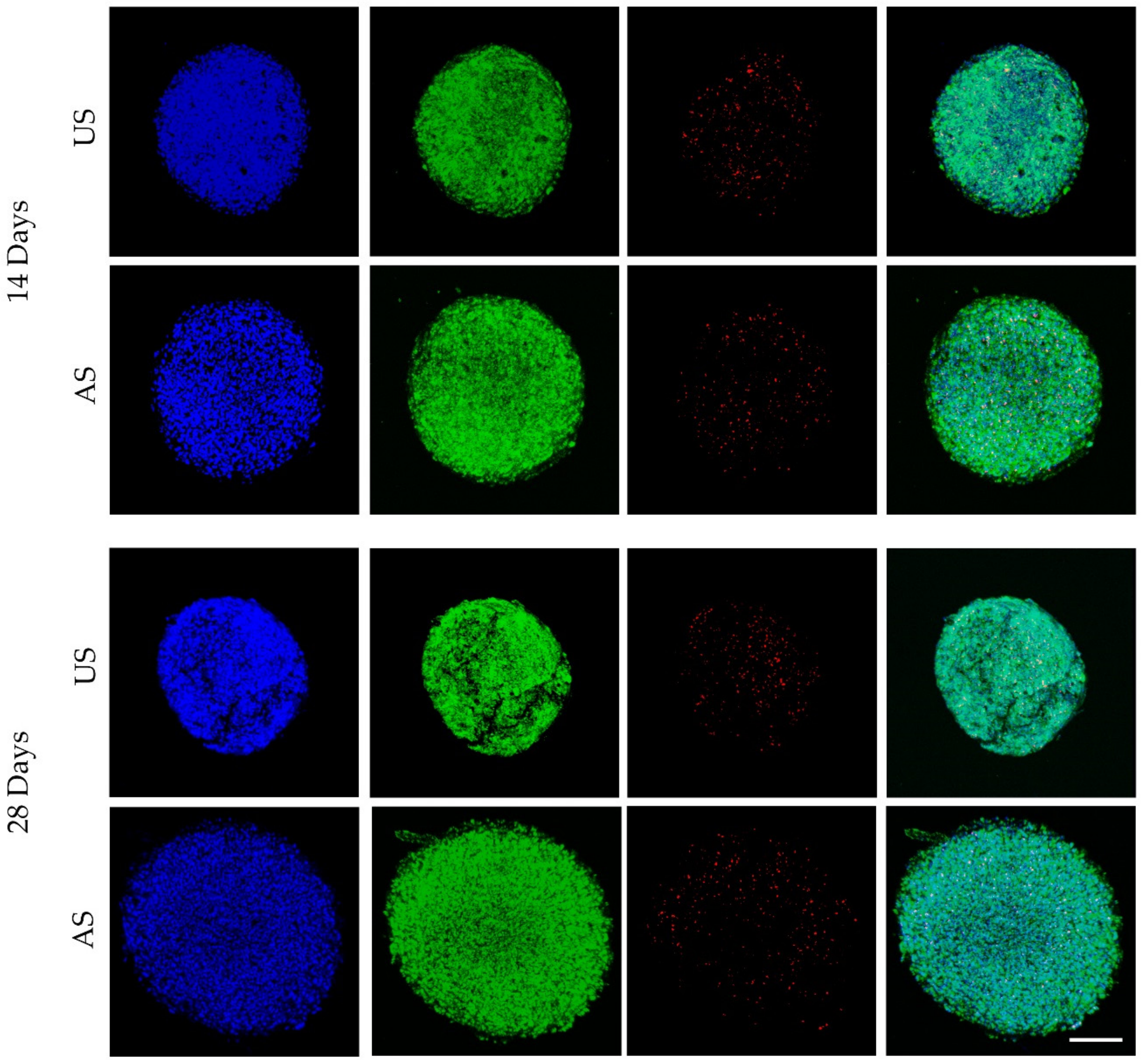

Figure S1: Live/dead-staining of unstimulated and adipogenically stimulated 3D spheroids after 14 and 28 days (n = 4, LSM 780, Zen black software, overlay of z-stack, green: live, red: dead, blue: nuclei; scale bar: 200 µm).

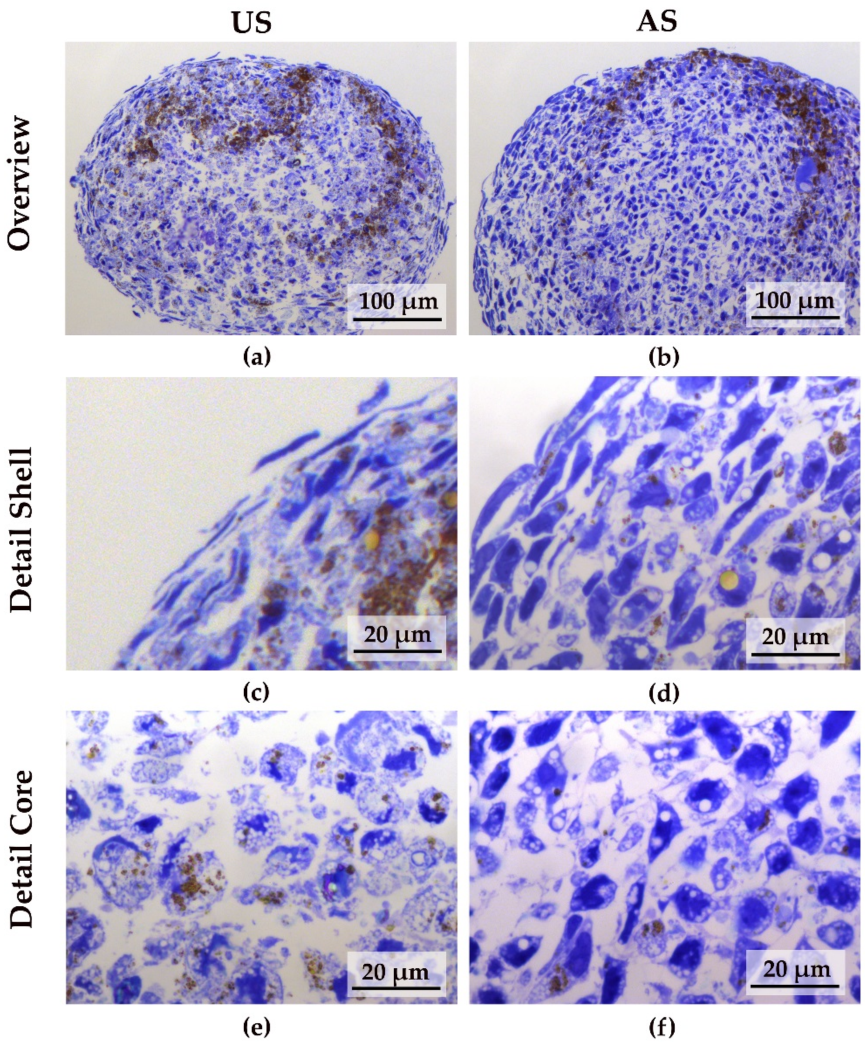

Figure S2: Thin sections (0.5 µm) of unstimulated and adipogenically stimulated 3D spheroids after 28 days of culture. Overview images of (a) an unstimulated (US) spheroid and (b) an adipogenically (AS) stimulated spheroid (scale bars: 100 µm) (c) detail of the shell region of an unstimulated spheroid, (d) detail of the shell region of an adipogenically stimulated spheroid, (e) detail of the core of an unstimulated spheroid, (f) detail of the core region of an adipogenically stimulated spheroid (scale bars: 20 µm; representative images of 3 experiments; light microscopy (Zeiss Axioskop 40), toluidine blue staining).
